# Rethinking diuretic use in acute kidney injury: effective prevention or false hope?

**DOI:** 10.1186/s40560-025-00804-z

**Published:** 2025-06-16

**Authors:** Hiroyuki Yamada, Maki Murata, Hiroyuki Hashimoto

**Affiliations:** 1 Department of Primary Care and Emergency, Graduate School of Medicine, Kyoto University, 54 Shogoin-Kawaharacho, Sakyo-Ku, Kyoto, 6068507 Japan; 2 Department of Nephrology, Graduate School of Medicine, Kyoto University, Kyoto, Japan; 3 Department of Emergency Medicine and Critical Care, National Hospital Organization Kyoto Medical Center, Kyoto, Japan; 4 Department of Pharmacoepidemiology, Kyoto University, Kyoto, Japan

## Abstract

A recent meta-analysis of randomized controlled trials (with pre-specified methods and the largest sample to date) found that prophylactic diuretics significantly reduce acute kidney injury (AKI) incidence in high-risk patients but provide no benefit in treating established AKI. These findings challenge current AKI management guidelines, which generally discourage prophylactic diuretic use. However, given heterogeneity among trials and some risk of bias, caution is advised before generally altering clinical practice until further research confirms these findings.

## Comment

Clinical practice acute kidney injury (AKI) guidelines have long discouraged the use of prophylactic diuretics in at-risk patients [1, 2]. This stance reflects consistent trial evidence that loop diuretics confer no improvement in key outcomes and may even cause harm. Multiple randomized trials and meta-analyses have shown no significant reduction in AKI incidence, need for dialysis, or mortality with prophylactic diuretics [1]. Indeed, one randomized controlled trial (RCT) reported a higher rate of postoperative renal dysfunction in patients administered furosemide than in controls (14.6 vs. 0%) [1, 3]. Furthermore, any theoretical benefit of increased urine output is believed to be outweighed by the risk of diuretic-induced hypovolemia and hypotension, which can exacerbate renal hypoperfusion and injury [4]. Given this lack of proven benefits and potential for harm, expert panels have traditionally advised against the use of diuretics to prevent AKI [1, 2].

A recent meta-analysis of RCT conducted by our team examined the preventive and therapeutic effects of diuretics on AKI [5] . This comprehensive review included 64 RCTs (*n* = 9871) encompassing a wide range of diuretic agents and clinical scenarios. The majority of trials evaluated loop diuretics, natriuretic peptides, or renal guard system, together accounting for most of the evidence. These findings indicate that while diuretics are not effective for treating AKI, they show promise in preventing AKI, even in high-risk situations. Namely, this implies that diuretic administration should occur prior to kidney damage to decrease the need for kidney replacement therapy and lower mortality rates.

Our meta-analysis, which utilized a pre-specified methodology and included a substantially larger sample size than previous studies, represents a significant contribution to this field of research. Meanwhile, it is important to note that these results contradict the existing AKI management guidelines [1, 2] . These guidelines generally do not endorse diuretics for AKI prevention, except in cases of volume overload. Our meta-analysis challenges these recommendations due to its comprehensive sample size and methodological approach. Recent studies on diuretic medications, such as sodium-glucose transport proteins 2 inhibitors and angiotensin receptor-neprilysin inhibitor, have also demonstrated their kidney-protective properties [6, 7].

However, several factors suggest caution before generally altering clinical practices to proactive diuretic administration for AKI prevention. First, the meta-analysis comprehensively extracted RCTs with various clinical contexts. Although the overall risk ratio could be advantageous for AKI prevention, our subgroup analysis showed that the advantage effect fluctuated depending on the clinical context. Other than perioperative or cardiac-related interventions patients minimally benefit from progressive diuretics administration. Second, all the included RCTs were evaluated using the Risk of Bias 2 tool. Most of them were assessed as having some concerns, particularly due to deviations from intended interventions and selection of the reported results, necessitating careful interpretation of the combined results. Lastly, our subgroup analysis revealed that natriuretic peptides showed the most significant AKI prevention effects, whereas other diuretics did not. This suggests that the observed AKI prevention might be attributed to the specific pharmacological effects of natriuretic peptides or insufficient sample size of other diuretics.

Given these considerations, the authors of this study are even hesitant to definitively recommend widespread diuretic administration in clinical practice for AKI prevention. Additional research is necessary to determine whether these findings truly represent a new avenue for using diuretics to prevent AKI.

## Data Availability

No datasets were generated or analysed during the current study.
